# The Association between Anxiety and/or Depression Symptoms with Gastrointestinal Complaints Five Years after Metabolic and Bariatric Surgery - a Cross-sectional Study

**DOI:** 10.1007/s11695-026-08899-5

**Published:** 2026-09-21

**Authors:** Nadia Botros, Yonta G.R. van der Burgh, Laura N. Deden, Elske M. van den Berg, Agnes A.M. Berendsen, Eric J. Hazebroek

**Affiliations:** 1 Vitalys Obesity Clinic, part of Rijnstate hospital, Arnhem, Netherlands; 2 Wageningen University & Research, Wageningen, Netherlands; 3 Novarum Center for Eating Disorders and Obesity, Amstelveen, Netherlands

**Keywords:** Metabolic and bariatric surgery, Anxiety, Depression, Gastrointestinal complaints

## Abstract

**Introduction:**

Gastrointestinal (GI) symptoms are common following metabolic and bariatric surgery (MBS) and can significantly impair quality of life. Previous research indicates that anxiety and depressive symptoms could be associated with GI complaints. This study aims to investigate the association between anxiety and/or depressive symptoms and GI complaints five years after MBS.

**Methods:**

In this cross-sectional study 220 participants who underwent MBS were included. Self-reported anxiety and/or depressive symptoms were measured with the *Hospital Anxiety and Depression Scale* and GI complaints with the *Gastrointestinal Symptom Rating Scale*. Differences in GI complaint scores between participants with no, mild or severe anxiety and/or depression symptoms were assessed using *Kruskal-Wallis* and post-hoc tests. Furthermore, the association between anxiety and/or depressive symptom categories and moderately-severe to severe GI complaints (any GRSR item score ≥ 5 out of 7) was assessed by modified *Cox* regression.

**Results:**

Participants were on average 50 ± 11 years of age five years post-MBS, mostly female (81%) and predominantly underwent RYGB (87%). 28% reported anxiety and/or depressive symptoms, of which 15% mild and 13% severe. 42% of participants reported moderately-severe to severe GI complaints, of which 20% was severe. Participants with severe anxiety and/or depressive symptoms had significantly higher total and domain GI complaint scores (abdominal pain, reflux syndrome, constipation syndrome) than participants without these symptoms (*p*<.001). Similar trends were found in participants with mild anxiety and/or depressive symptoms (total *p*<.001, reflux syndrome *p*=.048, constipation syndrome *p*=.005), the association with abdominal pain was on the borderline of significance (*p*=.05). Participants with mild or severe anxiety and/or depressive symptoms respectively had a 1.75 (95%CI 0.98–3.11; not statistically significant) and 2.55 (95%CI 1.45–4.47) times higher risk (BMI and sleep adjusted prevalence ratio’s) of experiencing moderately-severe to severe GI complaints compared to those without.

**Conclusion:**

Participants 5-years post-MBS with mild to severe self-reported anxiety and/or depressive symptoms are at a higher risk of reporting more severe GI complaints. These findings underscore the need to assess and refer anxiety and/or depressive symptoms when counselling patients after MBS with GI complaints.

**Supplementary Information:**

The online version contains supplementary material available at https://doi.org/10.1007/s11695-026-08899-5.

## Introduction

Patients undergoing metabolic and bariatric surgery (MBS) commonly experience anxiety and depressive symptoms, which initially tend to diminish postoperatively [1–4]. The prevalence of anxiety symptoms decreased from 25% before MBS to 17% after surgery, according to a review of studies with a mean follow-up time of 34 months [2]. Similarly, depressive symptoms decreased from 35% preoperative to 20% postoperatively [2]. However, on the longer term the severity of anxiety and depressive symptoms eventually seem to (partially) return to pre-surgery levels [5–7].

An important postoperative outcome that may be related to anxiety and depressive symptoms are gastrointestinal (GI) complaints. GI complaints are frequently reported after MBS, with studies estimating that 21–54% of patients experience these symptoms [8–10]. Anxiety and depressive symptoms have been positively associated with GI complaints, for example in general, primary care and irritable bowel syndrome (IBS) populations [11–14]. This relationship is potentially mediated through the gut-brain-microbiota axis [15, 16]. Only few studies have reported on the association between anxiety and/or depressive symptoms and GI complaints after MBS. Kvalem et al. (2020) for example partly described this association by investigating the somatic symptom burden of pain, fatigue, dumping, diarrhea, and vomiting [17]. They observed bidirectional positive associations at both one and three years after MBS [17].

Although anxiety and depressive symptoms and GI complaints are both prevalent in the longer term after MBS, their relationship in this periodremains poorly understood. Existing evidence is limited, particularly regarding a broader range of GI complaint outcomes and on the longer term. A better understanding of this association may support the identification of patients who could benefit from more targeted postoperative care and guide future research aimed at optimizing long-term management after MBS. This eventually may improve the patients’ quality of life, postoperative satisfaction and decrease health care use.

To further address this gap, a cross-sectional study was performed on the association between self-reported anxiety and/or depressive symptoms (no, mild, severe) and GI complaints assessed both as complaint scores (total, abdominal pain, gastroesophageal reflux, diarrhea, indigestion, constipation) and as the risk of experiencing moderately-severe to severe GI complaints five years after MBS. This analysis was part of a larger cross-sectional observational study on the quality of life five years after MBS.

## Methods

### Study Design and Population

All patients aged 18 to 70 years who underwent Roux-en-Y gastric bypass (RYGB), sleeve gastrectomy (SG), or a revisional procedure (e.g. RYGB following gastric band) in 2015 were invited in 2020 to complete 16 online questionnaires as part of the QUINT study, a cross-sectional observational study on quality of life including GI complaints and mental health. Exclusion criteria were insufficient command of the Dutch language or other MBS in 2015 (such as acute surgery, or other revisions, e.g. distalisation or Single Anastomosis Duodenal Ileal bypass or gastric bypass reversal). For this specific analysis an additional exclusion criterium was not being able to compute Hospital Anxiety and Depression Scale (HADS) or Gastrointestinal Symptom Rating Scale (GSRS) scores due to missing values. All eligible participants were invited via email and received a reminder email after 5 days and 3 weeks. The study procedures were approved by the institutional research committee of [HOSPITAL]. All participants provided informed consent for participating in this study.

Of all 1,037 patients who underwent RYGB, SG or a revisional procedure in 2015, 377 (35%) gave consent for participating in the study. Of these patients, 233 (62%) filled in both the HADS questionnaire and the GSRS questionnaire. Thirteen participants were excluded as it was not possible to compute an anxiety/depression score due to missing values. This resulted in a total sample of 220 included participants in the current study.

### Data Collection

#### Participant Characteristics

The following participant characteristics were retrieved from self-reported online questionnaires five years post MBS: general/sociodemographic characteristics (age in years, gender, Body Mass Index (BMI, kg/m^2^) five-year total body weight loss percent (%TWL), education type, work status, financial situation); lifestyle factors (smoking (yes/no), alcohol use (yes/no), sleep (hours per night), physical activity (hours per week estimated using the SQUASH questionnaire [18]), self-reported current medical conditions (yes/no: diabetes mellitus, cardiovascular disease, mental health problems, bowel disorders (inflammatory bowel disease (IBD); irritable bowel syndrome (IBS); gastroesophageal reflux disease (GERD); lactose intolerance, celiac disease)). If missing from questionnaires, body weight and height data were retrieved from medical files if there was consent for this. The anthropometric measurements were used to calculate BMI and five-year %TWL: weight loss at five years/preoperative weight * 100).

#### Anxiety and Depressive Symptoms

The validated Dutch version of the *Hospital Anxiety and Depression Scale* (HADS) questionnaire was used to assess anxiety and depressive symptoms [19, 20]. The HADS consists of 14 items (score 0–3), with 7 items on anxiety, and 7 on depression (range sum score per subscale: 0–21; Cronbach’s alpha coefficient for general medical patients 0.84 for both the anxiety and depression subscale) [19, 20]. Sum scores per subscale were classified into the following categories: no symptoms (< 8), mild symptoms (8–10), and severe symptoms (≥ 11). As anxiety and depressive symptoms often co-occur and are difficult to differentiate by the HADS [21], the anxiety and depression symptom categories were combined: e.g. if a participant reported anxiety symptoms, *or* depressive symptoms, *or* both, the participant was classified to have anxiety *and/or* depressive symptoms.

#### Gastrointestinal Symptoms

To which extent GI-problems were present in the past week, was measured by the validated *Gastrointestinal Symptom Rating Scale* (GSRS) [22]. The GSRS is a 15-item questionnaire, in which items are scored on a scale ranging from 1 (‘no discomfort’) to 7 (‘severe discomfort’). Symptoms were classified into five domains: abdominal pain, gastroesophageal reflux, diarrhoea, indigestion, and constipation. Per domain the average score was calculated. The total GSRS-score was calculated as the average of the 15 item scores.

### Data Analysis

Participant characteristics, in total and stratified for anxiety and/or depressive symptoms, are shown as means ± SD for continuous data, medians [P25; P75] for non-normally distributed data, or n (%) for categorical data. Differences in participant characteristics between anxiety and/or depressive symptoms categories were determined with *χ*^*2*^ or *Fisher’s exact tests* for categorical variables, and *ANOVA tests* for continuous variables or *Kruskal Wallis* tests if data was not normally distributed.

Differences in GI complaints across levels of anxiety and/or depressive symptoms (no, mild, or severe) were determined using *Kruskal Wallis* tests as these scores did not follow a normal distribution. Dunn post-hoc tests were done to determine which groups were different from each other, using the *Benjamini-Hochberg* method for adjusting *p*-values for multiple comparisons.

The association between anxiety and/or depressive symptoms and GI complaint severity levels was determined by means of a modified *Cox* regression analysis with a constant time variable (t = 1), giving prevalence ratio’s (PR) and their 95% CI’s. A modified *Cox* regression analysis was chosen as it gives more conservative, better interpretable results compared to logistic regression in cross-sectional studies [23]. Having no anxiety and/or depression symptoms was used as the reference group. GI complaints were grouped into two levels: none to moderate GI complaints (any of the 15 GRSR item score < 5 out of 7), and moderately severe to very severe GI complaints (any GRSR item score ≥ 5 out of 7). Assessed potential confounders and covariates were age, BMI, hours of sleep and physical activity. Due to low subgroup participant numbers, it was not possible to include gender and surgery type. BMI and hours of sleep slightly changed the estimates and were added to the model to improve estimate precision. This analysis was performed in 200 participants due to missing values in BMI and hours of sleep.

Missing questionnaire data was handled in the following manner: if a participant had less than 50% missing data for calculating an average GSRS domain score, the mean score of the non-missing domain items was imputed (*n* = 8). If the sum score for the HADS could not be calculated due to missing values, participants were excluded for this analysis (*n* = 13).

Data was analysed using R and RStudio (RStudio, Inc., Boston, version 2023.12.1.402, and R version 4.3.0, R foundation, 2020, Vienna, Austria). *P*-values < 0.05 were considered as statistically significant.

## Results

### Study Participants

Participants were on average 50 ± 11 years of age, were mostly female (81%) and predominantly underwent Roux-en-Y gastric bypass (87%). Participant characteristics are shown in Table 1.

**Table 1:** Participant characteristics of 220 participants 5 years after MBS and stratified by anxiety and/or depression symptoms

	Overall (*N* = 220)	HADS Anxiety and/or depression symptoms			
		No (*n* = 159)	Mild (*n* = 33)	Severe (*n* = 28)	
HADS anxiety score	4 [1; 7]	2 [1; 4]	9 [7; 10]	12.5 [11; 15]	
HADS depression score	2 [0; 6]	1 [0; 3]	7 [3; 8]	10.5 [7; 14.3]	
General/sociodemographic					***P-*** **value**
Age (years)	49.8 ± 11.4	50.6 ± 11.2	50.5 ± 10.9	44.3 ± 11.7	***0.035***
Gender: female	178 (81%)	126 (79%)	30 (91%)	22 (79%)	0.303
Pre-operative BMI (kg/m ^2^)	45.3 ± 6.9	45.0 ± 6.4	44.2 ± 6.5	48.4 ± 9.0	0.131
BMI (kg/m^2^) ^B^	31.1 ± 5.8	30.5 ± 5.6	32.3 ± 5.3	33.6 ± 7.0	***0.036***
%TWL at 5 years ^B^	30.8 ± 11.3	32.0 ± 10.3	26.2 ± 12.5	29.6 ± 13.9	0.054
Surgery type					***0.026***
RYGB	159 (72%)	121 (76%)	21 (64%)	17 (61%)	
GS	29 (13%)	21 (13%)	2 (6%)	6 (21%)	
REDO (to RYGB)	32 (15%)	17 (11%)	10 (30%)	5 (18%)	
Education type ^F^					0.290
Primary	12 (6%)	7 (4%)	3 (9%)	2 (7%)	
Vocational	140 (64%)	98 (62%)	20 (61%)	22 (79%)	
Theoretical/higher vocational	58 (26%)	46 (29%)	8 (24%)	4 (14%)	
Work status ^D^					0.085
Salaried/self-employed	147 (67%)	108 (68%)	24 (73%)	15 (54%)	
Unemployed/disabled/illness	33 (15%)	21 (13%)	3 (9%)	9 (32%)	
Retired	17 (8%)	15 (9%)	1 (3%)	1 (4%)	
Other	16 (7%)	9 (6%)	4 (12%)	3 (11%)	
Financial situation ^D^					0.065
Some – severe problems	26 (12%)	14 (9%)	3 (9%)	9 (32%)	
I get by financially	48 (22%)	34 (21%)	10 (30%)	4 (14%)	
I get by well/excellent	125 (57%)	93 (59%)	19 (58%)	13 (46%)	
Not specified	14 (6%)	11 (7%)	1 (3%)	2 (7%)	
Lifestyle factors					
Smoking: yes ^A^	39 (18%)	28 (18%)	5 (15%)	6 (21%)	0.747
Alcohol use: yes ^G^	91 (41%)	70 (44%)	14 (42%)	7 (25%)	0.130
Sleep (hours per night) ^H^	6.6 ± 1.4	6.9 ± 1.2	6.1 ± 1.5	5.8 ± 1.6	***< 0.001***
Physical activity (hours per week) ^C^	40 [22; 62]	44 [27; 62]	34 [11; 47]	28 [16; 53]	***0.014***
Self-reported medical conditions					
CVD ^D^	29 (13%)	21 (13%)	5 (15%)	3 (11%)	0.903
DM	30 (14%)	26 (16%)	2 (6%)	2 (7%)	0.209
Bowel disorders/complaints ^F^	33 (15%)	18 (11%)	6 (18%)	9 (32%)	***0.025***
IBS	14 (6%)	7 (4%)	3 (9%)	4 (14%)	
IBD	6 (3%)	4 (3%)	1 (3%)	1 (4%)	
Lactose intolerance	5 (3%)	4 (3%)	1 (3%)	0 (0%)	
GERD	11 (5%)	6 (4%)	1 (3%)	4 (14%)	
Mental health problems* ^E^	27 (12%)	3 (2%)	7 (2%)	17 (61%)	***< 0.001***
Depression	15 (7%)	1 (1%)	2 (6%)	12 (43%)	
Anxiety	8 (4%)	0 (0%)	1 (3%)	7 (25%)	
Other/unspecified	11 (5%)	2 (1%)	4 (2%)	5 (2%)	
GI complaints					
Moderately severe to severe GI complaints**	93 (42%)	54 (34%)	19 (58%)	20 (71%)	***< 0.001***
Severe GI complaints***	43 (20%)	20 (13%)	10 (30%)	13 (46%)	***< 0.001***

Variables are presented as mean ± SD, median [P25; P75], or n (%)

HADS: Hospital Anxiety and Depression Scale, BMI: Body Mass Index, %TWL: percentage total weight loss, CVD: Cardiovascular disease, DM: Diabetes Mellitus, IBS: irritable bowel syndrome, IBD: inflammatory bowel disorder, GERD: gastroesophageal reflux disease, GI: gastrointestinal

* Self-reported mental health problems or disorders. Other: includes for example Attention Deficit Hyperactivity Disorder, eating disorders and personality disorders. Unspecified: prevalence of a mental health problem was indicated, but its nature was not further specified by the participant

** Any GSRS (Gastrointestinal Symptom Rating Scale) item score ≥ 5 out of 7

*** Any GSRS item score ≥ 6 out of 7

Number of missings: A: 3, B: 4, C: 5, D: 7, E: 9, F: 10, G: 12, H: 17

Of the 220 participants, 61 (28%) reported anxiety and/or depressive symptoms. Of these 61 participants, 33 (15%) reported mild anxiety and/or depressive symptoms. Twenty-eight (13%) reported severe anxiety and/or depressive symptoms (see Supplementary Table 1 for more details). Of the 14 participants with severe depressive symptoms, 11 also had severe anxiety symptoms. Twenty-seven (12%) of the 220 participants reported to have a current mental disorder. These were mostly depressive (*n* = 15) or anxiety (*n* = 8) disorders (both: *n* = 5). Hundred-and-fifty-nine (72%) reported no anxiety and/or depressive symptoms. Compared to participants without symptoms, those with mild or severe anxiety and/or depressive symptoms were younger (severe symptom group only), had a slightly higher postoperative BMI, differed in surgical procedure type (less often primary RYGB), more often reported bowel disorders or symptoms, reported fewer hours of sleep per night and less hours of psychical activity per week. Participants with anxiety and/or depressive symptoms were more often dissatisfied with surgery results. In the severe anxiety and/or depression-group, 11 of the 28 patients (39%) were dissatisfied, of which 7 were due to complaints.

Of the 220 participants, 93 (42%) experienced any moderately-severe to severe GI complaint (score ≥ 5 out of 7). Of those 93 participants, 43 (20% of all participants) indicated experiencing any severe or worse GI complaint (score ≥ 6 out of 7). Only two out of the 220 participants reported any GI complaint to be very severe (score of 7 out of 7).

### Differences in GI Complaint Scores Across Anxiety and/or Depressive Symptom Levels

**Fig. 1:**
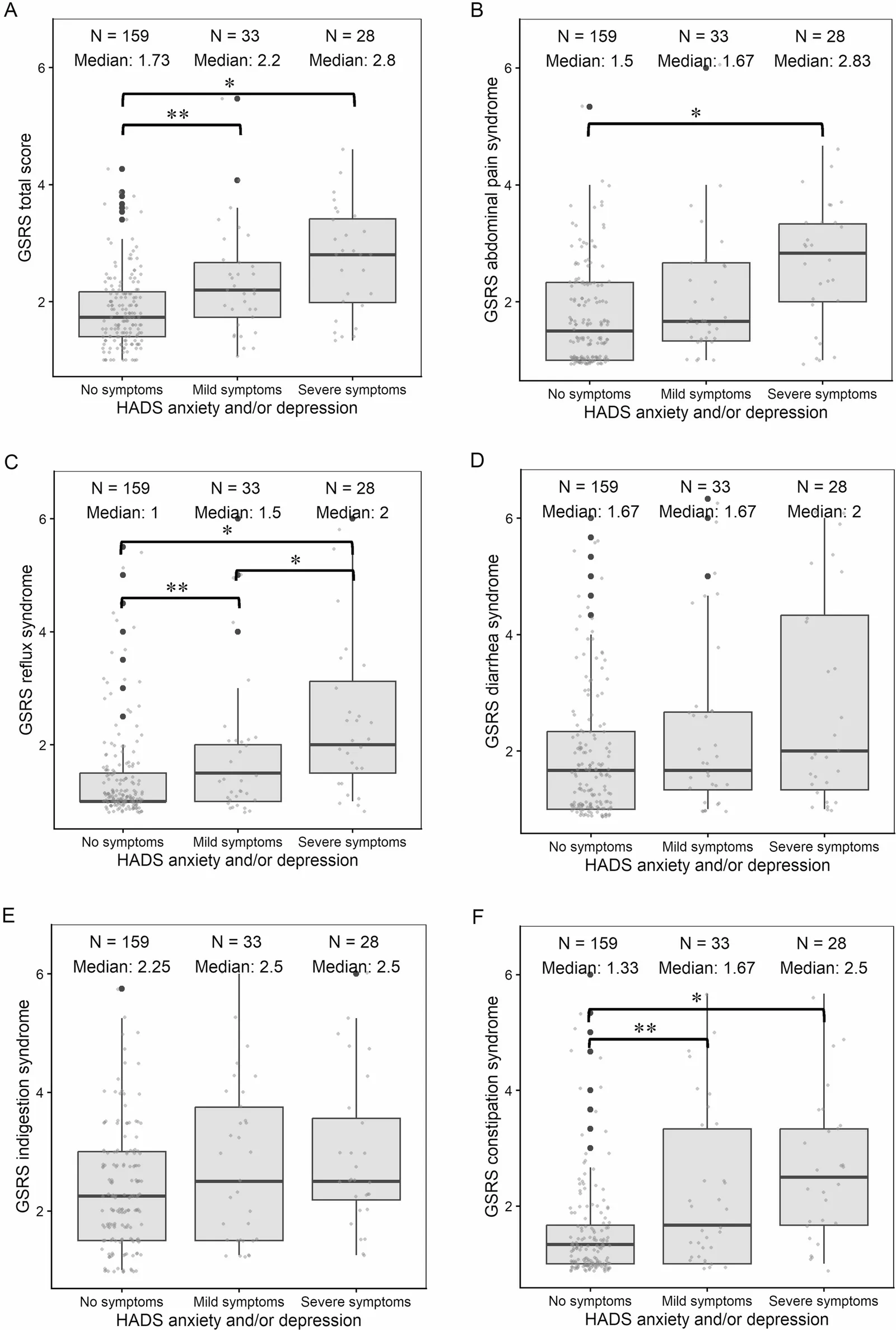
Boxplots of GI total and domain scores by anxiety and/or depression symptoms in 220 participants 5 years after MBS

Total and domain GRSR scores were significantly different between participants with no, mild or severe anxiety and/or depressive symptoms, showing positive trends (total, abdominal pain, reflux syndrome, constipation syndrome *P*<.001, diarrhea syndrome *p*=.028 and indigestion syndrome *P*=.039). Figure 1 shows boxplots of GI complaint scores by anxiety and/or depressive symptoms.

Post-hoc tests show that participants with *severe* symptoms had significantly higher total GI complaint scores (*p*<.001) and some domain scores than participants without anxiety and/or depressive symptoms (abdominal pain *p*<.001, reflux syndrome *p*<.001, constipation syndrome *p*<.001). Also, participants with *mild* anxiety and/or depressive symptoms had significantly higher GI complaint scores (total *p*<.001, reflux syndrome *p*=.048, constipation syndrome *p*=.005) than patients without anxiety and/or depressive symptoms. For abdominal pain this association was borderline significant (*p*=.05). Additionally, for reflux syndrome participants with *severe* anxiety and/or depressive symptoms had a significantly higher score than those with *mild* symptoms (*p*=.018). For diarrhea and indigestion syndrome none of the post-hoc comparisons were statistically significant after adjustment for multiple comparisons.

All Kruskal-Wallis tests indicated significant group differences in anxiety and/or depression levels. Post hoc test results are indicated within the figures: * *P*<.001, ** *P*<.05.

### Association of Anxiety and/or Depressive Symptoms with GI Complaint Severity Levels

Participants with mild anxiety and/or depressive symptoms tended to have a 1.75 (adjusted PR, 95% CI: 0.98–3.11) times higher risk of experiencing any moderately-severe to severe GI complaint compared those without anxiety and/or depressive symptoms (not statistically significant). For severe anxiety and/or depressive symptoms this risk was 2.55 (adjusted PR, 95% CI:1.45–4.47) times higher. Results of the modified Cox regression are shown in Table 2.

**Table 2:** Prevalence ratio’s (PR (95%CI)) for experiencing any moderately severe to severe GI complaints by anxiety and/or depression symptoms levels from modified Cox regression in 200* participants 5 years post- MBS

	HADS Anxiety and/or depression symptoms		
	No (*n* = 144)	Mild (*n* = 29)	Severe (*n* = 27)
Crude	**1.00**	1.80 (1.03–3.16)	2.61 (1.54–4.43)
Model 1 adjusted for BMI 5 years post-MBS	**1.00**	1.82 (1.03–3.19)	2.67 (1.56–4.59)
Model 2 additionally adjusted for hours of sleep	**1.00**	1.75 (0.98–3.11)	2.55 (1.45–4.47)

* 20 participants were excluded from this analysis due to missing values for BMI 5 years post-MBS and hours of sleep per night

## Discussion

This study aimed to explore the relationship between anxiety and/or depressive symptoms and GI complaints five years after MBS. The results indicated that participants with severe anxiety and/or depressive symptoms more often had moderately-severe to severe GI complaints compared to participants with no anxiety and/or depressive symptoms. They also had significantly higher total and GI complaint domain scores for reflux syndrome, constipation syndrome and abdominal pain. Participants with mild anxiety and/or depressive symptoms also reported higher GI complaint scores and tended to more often experience moderately-severe to severe GI complaints, although the latter was not statistically significant after adjusting for BMI and hours of sleep.

In this study, 23% of participants reported mild or severe anxiety symptoms, a higher prevalence than observed in a study four years post-MBS (14%) [6] and more similar to pre-MBS estimates (25%) [2]. Mild or severe depressive symptoms were reported by 16% of our participants. This was similar to findings at four years post-MBS [6], and slightly lower than the 20% prevalence reported in a review with a mean follow-up of 34 months [2]. A Norwegian study reported a mean anxiety HADS subscale score of 4.4 ± 4.2 after RYGB and 5.0 ± 4.2 after GS, for the depression score this was 2.3 ± 3.0 after RYGB and 2.6 ± 3.4 after GS [24]. In our study population (all surgery types) medians were 4 [1; 7] for HADS anxiety and 2 [0; 6] for depression scores. Moderately-severe to severe GI complaints were reported by 42% of our study participants, which is within the range indicated by previous studies, despite heterogeneity in symptom definitions [8–10]. In a study in 249 patients two years after RYGB the median GRSR score was 2.19 [1.63; 2.88], and 1.75 [1.38–2.31] in unoperated controls with severe obesity [25]. In the current study the overall median GSRS score was 1.87 [1.46; 2.42], lower than reported in the post-RYGB group in the previously mentioned study. The GSRS scores in our study were largely similar to Norwegian studies two years after MBS [24, 26].

Few studies have reported on the association between anxiety and depressive symptoms and GI complaints after MBS [10, 17]. Kvalem et al. studied a partially similar association: they observed a positive bidirectional association between anxiety/depressive symptoms and the somatic symptom burden (pain, fatigue, dumping, diarrhea, and vomiting) in 169 patients at both one and three years after MBS [17]. Høgestøl et al. reported that anxiety as well as depression symptoms were positively associated with chronic abdominal pain five years after MBS in univariate analysis [10]. They reported that anxiety or depression symptoms were not among the significant predictors for chronic abdominal pain after including the pain catastrophizing scale score, average bodily pain, BMI and gender to the model. The authors note that in this study only few study participants experienced anxiety or depressive symptoms.

Although only few studies exist on the association of depression and anxiety symptoms with GI complaints post-MBS [10, 17], this association is frequently reported in observational studies in diverse populations (e.g. with IBS, IBD, and GERD), although the underlying pathophysiological mechanisms and causal pathways are not entirely clear [14, 16, 27–30]. However, there is evidence pointing towards a causal relationship, as a 12-year prospective cohort showed that higher levels of anxiety (but not depression) at baseline was a strong predictor of developing GI complaints [31]. Within the microbiota-gut-brain axis various bidirectional communication pathways have been described [15]. These include the vagus nerve, microbiota and their metabolites [15, 16],. A different gut microbiota composition has been related to physiological and pathological processes in conditions such as mental disorders, pain, anxiety, IBS and obesity [15]. Also, metabolites such as tryptophan and short-chain fatty acids (SCFAs) have been related to mental wellbeing [32, 33].

This study has several strengths: to our knowledge this is one of the few studies investigating anxiety and/or depressive symptoms in relation to GI complaints on the longer term after MBS. In this study a broad spectrum of GI complaints was assessed, as were three gradations of anxiety and/or depressive symptoms. Additionally, two approaches of analysing GI complaints were used: assessing total and domain symptom scores and a clinically relevant cut off taking moderately-severe to severe GI complaints into account. Furthermore, validated questionnaires were used.

Several limitations should be noted. First, the cross-sectional design did not include data on preoperative GI complaints and psychological distress and their change over time. Therefore, within participant changes could not be taken into account. Second, causality within the studied association is unknown and is expected to be bidirectional. Third, small groups of participants with anxiety and/or depressive symptoms did not allow further subgroup analyses (e.g. according to surgery type). This also disabled us to adjust for other potentially relevant factors, such as gender (too small subgroup n) and surgery type (too small subgroup n). Fourth, there was no data available on other relevant factors such as medication (e.g. psychotropic, antibiotics and proton pump inhibitors) and dietary/behavioural parameters (such as diet quality, fibre intake and adherence to dietary recommendations after MBS). Fifth, although anxiety and depression symptoms often co-occur, it was not possible to investigate separate associations for both types of symptoms as subgroups were too small. Sixth, data on anxiety or depressive symptoms were self-reported, which could result in bias compared to interview-based data.

Although evidence suggests a bidirectional association between depression and/or anxiety symptoms with GI complaints post-MBS is plausible, the present study underscores the necessity for larger prospective observational studies, which include adequate measurement of behavioural parameters and medication. These include the following factors known influence GI functioning and microbiota-gut-brain activity: a high-fibre plant-based diet, exercise, sleep, stress and relevant medication use [15, 34–37].

## Conclusion

Patients five years post-MBS with severe self-reported anxiety and/or depressive symptoms were at a higher risk of reporting moderately-severe to severe GI complaints and reported higher GI complaint scores. Patients with mild anxiety and/or depressive symptoms also reported higher GI complaint scores. This highlights the importance of attention for co-occurring psychological distress when counselling patients with GI complaints after MBS during clinical consultations. It also highlights the urgency to find strategies to optimize treatment options in patients with both GI complaints and psychological distress.

## Supplementary Information

Below is the link to the electronic supplementary material.

ESM 1 (DOCX 43.1 KB)

## Data Availability

The data supporting the findings of this study are not publicly available. Researchers interested in accessing the data may contact the corresponding authors’ research department to discuss reasonable requests for data access.
