# Supplementary material for: The Association between Anxiety and/or Depression Symptoms with Gastrointestinal Complaints Five Years after Metabolic and Bariatric Surgery - a Cross-sectional Study
Source: Obes Surg. 2026 Sep 21;36(10):5307–15. doi: 10.1007/s11695-026-08899-5 (PMC13645904; doi:10.1007/s11695-026-08899-5)
Supplement: Supplementary file 1 [file 11695_2026_8899_MOESM1_ESM.docx]

***Supplementary table 1.*** Anxiety and depression symptoms according to the Hospital Anxiety and Depression scale in 220 participants 5 years after MBS.

|  | **Anxiety and/or depression symptoms** | | | | |  |
| --- | --- | --- | --- | --- | --- | --- |
|  | **No** | **Mild** | | **Severe** | | **Overall** |
|  | **n=159** | **n=33** | | **n=28** | | **N=220** |
| **Anxiety symptoms** |  | |  | |  |  |
| No anxiety symptoms | 159 | | 9 (27%) | | 1 (4%) | 169 (77%) |
| Mild anxiety symptoms | 0 | | 24 (73%) | | 2 (7%) | 26 (12%) |
| Severe anxiety symptoms | 0 | | 0 | | 25 (89%) | 25 (11%) |
| **Depression symptoms** |  | |  | |  |  |
| No depression symptoms | 159 | | 18 (55%) | | 8 (29%) | 185 (84%) |
| Mild depression symptoms | 0 | | 15 (46%) | | 6 (21%) | 21 (10%) |
| Severe depression symptoms | 0 | | 0 | | 14 (50%) | 14 (6%) |
